# Glucose dysregulation in antipsychotic-naive first-episode psychosis: in silico exploration of gene expression signatures

**DOI:** 10.1038/s41398-023-02716-8

**Published:** 2024-01-10

**Authors:** Jiwon Lee, Xiangning Xue, Emily Au, William B. McIntyre, Roshanak Asgariroozbehani, Kristoffer Panganiban, George C. Tseng, Maria Papoulias, Emily Smith, Jonathan Monteiro, Divia Shah, Kateryna Maksyutynska, Samantha Cavalier, Emril Radoncic, Femin Prasad, Sri Mahavir Agarwal, Robert Mccullumsmith, Zachary Freyberg, Ryan W. Logan, Margaret K. Hahn

**Affiliations:** 1 Institute of Medical Science, University of Toronto, Toronto, ON Canada; 2 Centre for Addiction and Mental Health, Toronto, ON Canada; 3 Department of Biostatistics, Graduate School of Public Health, University of Pittsburgh, Pittsburgh, PA USA; 4 Department of Pharmacology and Toxicology, University of Toronto, Toronto, ON Canada; 5 Department of Psychiatry, University of Pittsburgh, Pittsburgh, PA USA; 6 Department of Neurobiology, University of Massachusetts Chan Medical School, Worcester, MA USA; 7 Department of Neurosciences, University of Toledo, Toledo, OH USA; 8 ProMedica, Toledo, OH USA; 9 Department of Cell Biology, University of Pittsburgh, Pittsburgh, PA USA; 10 Department of Psychiatry, University of Massachusetts Chan Medical School, Worcester, MA USA; 11 Department of Pharmacology, Physiology & Biophysics, Boston University School of Medicine, Boston, MA USA; 12 Department of Psychiatry, University of Toronto, Toronto, ON Canada

**Keywords:** Pathogenesis, Schizophrenia

## Abstract

Antipsychotic (AP)-naive first-episode psychosis (FEP) patients display early dysglycemia, including insulin resistance and prediabetes. Metabolic dysregulation may therefore be intrinsic to psychosis spectrum disorders (PSDs), independent of the metabolic effects of APs. However, the potential biological pathways that overlap between PSDs and dysglycemic states remain to be identified. Using meta-analytic approaches of transcriptomic datasets, we investigated whether AP-naive FEP patients share overlapping gene expression signatures with non-psychiatrically ill early dysglycemia individuals. We meta-analyzed peripheral transcriptomic datasets of AP-naive FEP patients and non-psychiatrically ill early dysglycemia subjects to identify common gene expression signatures. Common signatures underwent pathway enrichment analysis and were then used to identify potential new pharmacological compounds via Integrative Library of Integrated Network-Based Cellular Signatures (iLINCS). Our search results yielded 5 AP-naive FEP studies and 4 early dysglycemia studies which met inclusion criteria. We discovered that AP-naive FEP and non-psychiatrically ill subjects exhibiting early dysglycemia shared 221 common signatures, which were enriched for pathways related to endoplasmic reticulum stress and abnormal brain energetics. Nine FDA-approved drugs were identified as potential drug treatments, of which the antidiabetic metformin, the first-line treatment for type 2 diabetes, has evidence to attenuate metabolic dysfunction in PSDs. Taken together, our findings support shared gene expression changes and biological pathways associating PSDs with dysglycemic disorders. These data suggest that the pathobiology of PSDs overlaps and potentially contributes to dysglycemia. Finally, we find that metformin may be a potential treatment for early metabolic dysfunction intrinsic to PSDs.

## Introduction

Individuals with psychosis spectrum disorders (PSDs), including schizophrenia, have a significantly higher risk of developing metabolic disorders such as type 2 diabetes (T2D) compared to the general population [1]. In turn, individuals with PSDs face increased cardiovascular mortality, resulting in decreased life expectancy by 20–25 years [2, 3]. Several factors contribute to metabolic abnormalities associated with PSDs, including antipsychotic (AP) drug exposure and illness-related lifestyle factors, such that metabolic outcomes worsen progressively with longer illness duration [1, 4]. However, extrinsic factors do not fully explain the metabolic risk present in PSDs. Abnormal glucose metabolism and insulin resistance are often already present in individuals who are at high-risk for PSDs or experiencing their first episode of psychosis (FEP), even prior to the introduction of APs [5–9]. Notably, schizophrenia confers a three-fold increase in risk for T2D. Indeed, AP-naive FEP has been associated with impaired glucose tolerance and insulin resistance, and as many as 15.4% of FEP patients in community settings have prediabetes [9, 10]. Furthermore, independent of additional risk factors, this schizophrenia-associated T2D risk is further exacerbated by beginning AP treatment [11]. This suggests that PSDs may present an intrinsic risk for metabolic dysfunction, foremost dysglycemia. Taken together, the pathobiological changes underlying PSDs may include biological pathways that not only contribute to illness psychopathology but may also explain premorbid or early dysglycemia.

Several genetic studies support causal biological associations between PSDs and dysglycemia. For instance, susceptibility genes for schizophrenia and T2D are found in overlapping biological networks, suggesting common underlying mechanisms [12]. Additionally, a prospective study found that genetic predisposition to T2D is associated with an increased risk of psychosis in young adulthood [13]. Further, a Mendelian randomization study demonstrated causal links between gene variants linked to elevated fasting insulin levels and schizophrenia risk [14]. Thus, changes at the gene level associated with PSDs may be responsible for dysglycemia among patients with PSDs, providing support for intrinsic dysglycemia in PSDs.

At the gene expression level, PSDs and T2D also show biological overlap [15]. However, it is unclear whether these overlapping gene expression changes can explain intrinsic dysglycemia in PSDs. Namely, this line of evidence has been based on work in AP-treated schizophrenia patients, precluding exclusion of extrinsic factors such as AP use. Additionally, examination of T2D gene expression signatures may lack external validity to early dysglycemia in FEP (*i.e.,* insulin resistance and prediabetic states such as impairments in fasting glucose and glucose tolerance), considering that individuals with FEP very rarely present with overt T2D [16]. Thus, examining gene expression of FEP patients who are AP-naive (lack prior AP exposure) enables us to study dysglycemia intrinsic to PSDs independently of potential confounds such as AP use and illness duration. Further, we posit that examining early dysglycemia may be more in line with the intrinsic disruptions in glucose metabolism observed in the early stages of psychosis.

Intriguingly, metabolic derangements early in the course of PSDs may be mechanistically linked to illness psychopathology, including treatment-resistant domains such as cognition. T2D and insulin resistance are associated with worse cognitive impairments in the general population and in schizophrenia [17, 18], which suggests that the pathophysiological mechanisms contributing to premorbid cognitive dysfunction and intrinsic dysglycemia in PSDs may overlap. Therefore, examining the overlap in gene expression between AP-naive FEP and non-psychiatrically ill unaffected individuals with dysglycemia may inform novel treatment options for both the psychiatric and metabolic aspects of PSDs.

Here, we investigated whether AP-naive FEP patients share overlapping gene expression patterns with non-psychiatrically ill individuals with early dysglycemia by meta-analyzing published transcriptomic datasets. We reasoned that the overlapping gene expression signatures between AP-naive FEP and early dysglycemia may represent gene expression changes endogenous to PSDs that are likely responsible for producing dysglycemia independent of extrinsic factors such as AP treatment. While post-mortem brain transcriptomics are rare in young AP-naive FEP patients, we used peripheral samples as an alternative avenue to pursue our investigations given significant transcriptomic overlaps in brain and peripheral blood [19, 20]. Additionally, data repositories were probed for peripheral transcriptomics of early dysglycemia subjects for comparison. The overlapping gene expression signatures between AP-naive FEP and early dysglycemia were analyzed for associated biological pathways and utilized to identify potential pharmacological treatments for intrinsic dysglycemia in PSDs. Taken together, our analyses revealed that PSDs and early dysglycemia share common gene expression changes that may be mediated by endoplasmic reticulum (ER) stress and abnormal brain bioenergetics. Further, the T2D drug metformin represents a potential treatment for intrinsic dysglycemia in PSDs.

## Methods

**Fig. 1:**
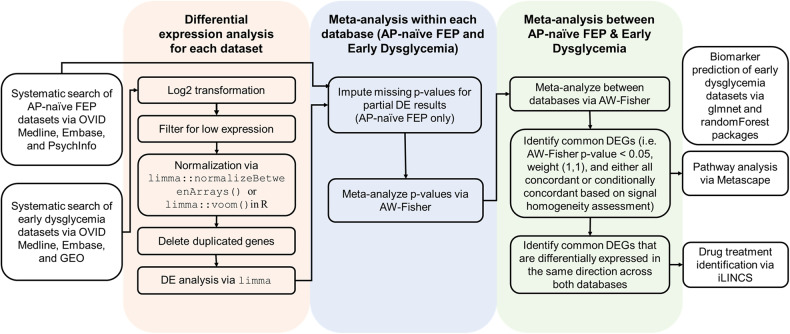
Study workflow. AP-naive FEP antipsychotic-naive first-episode psychosis, GEO Gene Expression Omnibus, DEG differentially expressed gene, iLINCS Library of Integrated Network-Based Cellular Signatures.

The workflow consisted of the following components: (1) systematic search and selection of relevant datasets; (2) differential expression analysis; (3) signal homogeneity assessment; (4) meta-analysis; (5) pathway enrichment analysis; (6) Integrative Library of Integrated Network-Based Cellular Signatures (iLINCS) connectivity analysis for identification of candidate drug treatments; and (7) biomarker prediction (Fig. 1).

### Antipsychotic-naive first-episode psychosis database

We employed a systematic search to curate transcriptomic datasets examining peripheral tissue of AP-naive FEP patients. Ovid PsychINFO, Embase, and Medline were searched for studies published before March 2021 (*PROSPERO* ID: 185602). The search string combined keywords covering three conceptual groups: AP-naive, psychosis, and transcriptomics (Table S1). Study de-duplication and selection took place in Covidence (https://www.covidence.org/). Each article was screened by two independent reviewers (JL, WBM, RA, and MP) based on title and abstract, followed by full texts according to pre-specified eligibility criteria. Inclusion criteria were: (1) case-control design; (2) lack of prior exposure AP exposure; (3) clinically confirmed FEP in cases; (4) adult age (18–65); and (5) use of transcriptomics approaches in the respective studies. Exclusion criteria included: (1) absence of an unaffected comparison group; (2) study examined too few genes (inferred from the range of p-values of the reported genes); or (3) co-morbidities or medications which may induce confounding gene expression changes (*e.g.,* kidney/liver disease, cancer, pregnancy, anti-inflammatory medications, and immunosuppressants). Disagreements were resolved between the two reviewers and involved a third reviewer (MKH) whenever necessary.

As only one of the five included studies provided raw gene expression data, we extracted differential gene expression (DGE) data, comprising the gene symbol, log-fold-change, and p-value. If available, full DGE analysis results were extracted. Otherwise, partial DGE analysis results were extracted (*i.e*., studies that reported significant genes only). Efforts were made to contact authors of unpublished studies (*e.g*., conference abstracts) for additional data.

### Non-psychiatrically ill early dysglycemia database

Transcriptomic datasets examining blood-derived tissue in non-psychiatrically ill early dysglycemia patients were searched systematically via OVID Embase, Medline, and Gene Expression Omnibus (GEO). The search in GEO was conducted in July 2020 and updated in March 2021 and again in March 2022. The search in Embase and Medline were conducted in March 2022. All searches were limited to human studies. The search string combined keywords comprising two conceptual groups: early dysglycemia and transcriptomics (Table S2). Each study was screened by two independent reviewers (EA, JL, ES, WBM, DS, KM, SC, ER, and FP) according to pre-specified inclusion criteria. Inclusion criteria were: (1) case-control design; (2) cases demonstrated prediabetic markers as defined by American Diabetes Association diagnostic criteria [21] (HbA1c: 5.7–6.4%, fasting plasma glucose: 100–125 mg/dl; or an oral glucose tolerance test 2 h glucose level: 140–199 mg/dl) or Homeostatic Model Assessment for Insulin Resistance (HOMA-IR) greater than 1.8 [22]; and (3) the study examined transcriptomics of blood-derived tissue. The justification for the last inclusion criterion was to make the early dysglycemia datasets comparable to the AP-naive FEP studies, most of which examined blood-derived tissue. Datasets were excluded if the study examined: (1) non-human tissue; (2) patients with overt type 1 or 2 diabetes; (3) psychiatrically-ill patients; (4) patients with co-morbidities or medications as deemed clinically significant which may induce confounding gene expression changes (*e.g*., kidney/liver disease, cancer, pregnancy, anti-inflammatory medications, and immunosuppressants); (5) lack of unaffected subjects used as a comparator; or (6) early dysglycemia cases and unaffected subjects were not matched for body mass index (BMI). The last criterion was applied to segregate glucose dysregulations occurring independently of adiposity. Raw gene expression data for all the included studies were available and downloaded from GEO.

### Differential expression analysis of non-psychiatrically ill early dysglycemia datasets

Differential expression analysis was conducted on the non-psychiatrically ill early dysglycemia datasets. The analytical pipeline consisted of the following: (1) log2 transformation; (2) filtering to remove genes with low expression; (3) quantile normalization in R; and (4) differential expression analysis using the limma package in R [23]. Log2 transformation was applied to datasets if one of the two following conditions were met: (1) the 99th quantile is greater than 100, or (2) the range of the data is greater than 50 and the first quartile is greater than 0. To filter for low expression, genes that have negative values before any transformation in more than half of the samples were filtered, except for genes that were present in the AP-naive FEP results. We then performed quantile normalization in R, as needed. For genes with duplicate records, we retained the record with the greatest variance. Subsequently, limma package in R [23] was used to identify differentially expressed genes (DEGs) for each study. One study [24] examined two contrasts with completely different participants, which were treated as two separate studies; two-group differential expression analysis was conducted on each contrast separately.

### Signal homogeneity assessment

To ensure comparability and homogeneity among the studies within each database (AP-naive FEP and non-psychiatrically ill individuals with early dysglycemia), we examined the concordance of differential expression signals by summarizing the number of genes that have the same direction of effect size across studies. We found that many genes with discordant directions of effect size possessed only marginal signals in some studies and could therefore be considered noise. To account for this, we introduced the conditional concordance criteria, where only studies with *p* < 0.05 are checked for concordance of signal directions. This ensured a focus on studies with more robust evidence for differential expression. Finally, genes that were not concordant in all studies or not conditionally concordant were considered discordant. In summary, we categorized all genes into three categories: (1) all concordant (ConSat = “C”), if the gene shows the same direction of differential expression across all studies with accessible effect size (because we do not have the full differential expression results from some FEP studies); (2) conditionally concordant (ConSat = “S”), if the gene shows the same direction of differential expression with *p* < 0.05 in all studies with accessible effect size (the *p* < 0.05 criteria is applied to filter out marginal signals); and 3) discordant (ConStat = “D”), if the gene does not satisfy the above two criteria.

### Meta-analysis

We first meta-analyzed studies within each database (AP-naive FEP and non-psychiatrically ill early dysglycemia). To meta-analyze the AP-naive FEP datasets, the truncated *p*-values combination method [25] was applied. Briefly, mean imputation was used to impute the missing *p*-values for datasets with partial differential expression results. Then, the *p*-values for all studies were combined using the adaptively weighted Fisher’s method (AW-Fisher) [26]. The early dysglycemia datasets were meta-analyzed by directly applying AW-Fisher, as they comprised of full DGE data only. The resulting meta-analyzed data from each database (AP-naive FEP and non-psychiatrically ill early dysglycemia) were then meta-analyzed with each other using AW-Fisher to identify common DEGs between AP-naive FEP and early dysglycemia. Common DEGs were defined as: (1) genes with AW-Fisher *p* < 0.05, weight (1,1); and (2) genes classified as either concordant or conditionally concordant for both AP-naive FEP and early dysglycemia by the signal homogeneity assessment. Additionally, log-fold-changes were combined by calculating a weighted average across studies that reported log-fold-changes for each gene, where the weight was based on the inverse p-value of each gene within each study.

### Pathway enrichment analysis by Metascape

Metascape, an annotation and analysis tool to examine gene expression data [27], was used for pathway analysis. The gene symbol and *p*-values of the common DEGs between AP-naive FEP and non-psychiatrically ill early dysglycemia were inputted into Metascape. Additionally, we inputted the full list of genes that overlapped across studies as background genes. The following parameters were applied in the analysis: minimum overlap of 3, minimum enrichment of 1, and *p* < 0.05. We applied the following pathway databases: GO Biological Processes, Reactome Gene Sets, and KEGG Pathway. Additionally, pathways with ≤5 gene members were excluded to avoid interpretation challenges associated with small pathways.

### Prediction efficiency of biomarkers

We used machine learning models to test whether genes identified from the meta-analysis demonstrate predictive accuracy as biomarkers for illness status. Because the raw data of the AP-naive FEP studies were largely unavailable, we only validated the possibility of predicting non-psychiatrically early dysglycemia status using our identified gene list. We accessed the raw data of the five early dysglycemia studies from GEO. After routine normalization in each study (as described earlier), we further centered and scaled the expression of each gene to reduce potential between-study heterogeneity. This resulted in gene expression data with a mean of 0 and a standard deviation of 1 for each gene in each study. Here, only genes that were identified as differentially expressed by the meta-analysis were inputted into the machine learning models.

Two well-recognized machine learning models were implemented for the prediction task: (1) the elastic net regularized generalized linear model (R package glmnet), and (2) random forest model (R package randomForest). To avoid overfitting, we used a cross-validation procedure to evaluate the performance of the models. We trained the model using data from four studies combined, and the prediction accuracy was assessed on the one study left as test data. This procedure was repeated until all five studies were used as the test data once, and we reported the final prediction accuracy as a mean of the five prediction accuracies.

We conducted the parameter tuning using nested cross-validation within the training data for the best prediction accuracy. For the glmnet model, we performed a grid search to tune the elastic net mixing parameter (α) and the regularization parameter (λ). For random forest model, we tuned number of trees (ntree) and the number of variables sampled for node split (mtry).

### Identification of drug treatments via iLINCS

We used iLINCS to identify pharmacological agents that have gene expression patterns discordant (reverse) to the common DEGs of AP-naive FEP and non-psychiatrically ill individuals with early dysglycemia. This approach represents a rational approach to identify novel pharmacological treatments for intrinsic dysglycemia in PSDs. Specifically, we reasoned that pharmacological agents with discordant gene expression patterns may be able to treat intrinsic dysglycemia in PSDs by correcting the common DEGs shared between AP-naive FEP and early dysglycemia. Because the “concordance score” of each pharmacological agent relies on the direction of dysregulation of each gene, we first selected common DEGs that were dysregulated in the same direction in both AP-naive FEP and early dysglycemia databases, based on the sign of the average log-fold change (upregulated and downregulated genes). The lists of upregulated and downregulated genes, containing the gene symbols, were inputted into ‘iLINCS Signatures’ for connectivity analysis. Connectivity analysis was conducted with the Connectivity Map signature signatures library, which contains gene expression profiles of various pharmacological perturbagens. Perturbagens were considered as potential treatments if they were highly discordant to the inputted signatures based on a previously used concordance score cutoff of ≤−0.321 [28].

### Scripts

All scripts used in the analysis are deposited in GitHub (https://github.com/XiangningXue/FEP_MetaAnalysis).

## Results

### Systematic search results

**Fig. 2:**
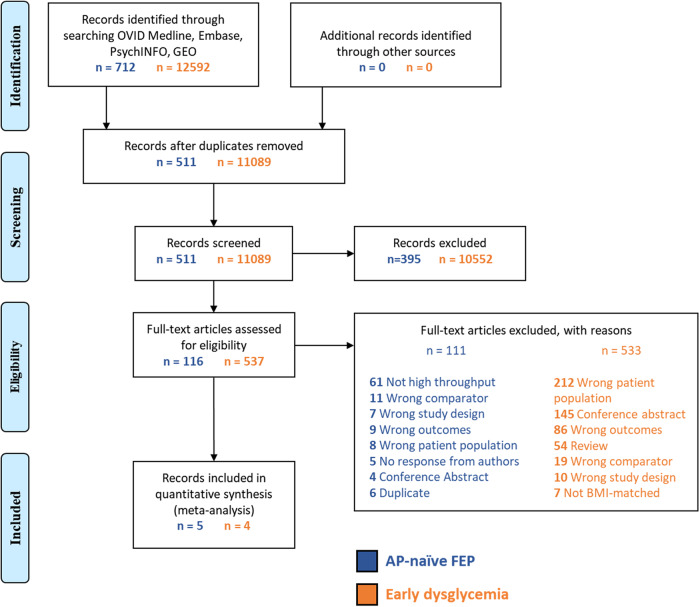
PRISMA flowchart. PRISMA flowchart of antipsychotic-naive first-episode psychosis (AP-naive FEP) and non-psychiatrically ill early dysglycemia studies.

The AP-naive FEP search identified 712 records, of which 5 met our inclusion and exclusion criteria and were included in subsequent analyses (Fig. 2) [29–33]. One study applied transcriptomics to two different tissue types [29], which were each treated as individual studies. Thus, a total of 6 datasets were retrieved and used in subsequent analyses. Five of these datasets applied microarrays, while the remaining dataset applied RNA sequencing for transcriptomic analysis. The datasets examined whole blood (*N* = 3), peripheral blood mononuclear cells (*N* = 1), fibroblasts (*N* = 1), and lymphoblastoid (*N* = 1) tissue. Further characteristics of included AP-naive FEP datasets are detailed in Table S3.

For early dysglycemia studies in non-psychiatrically ill subjects, of the 12,592 studies retrieved, 4 were included (GSE101931 [34], GSE21321 [35], GSE153837 [36], and GSE87005 [24]; Fig. 2), which contained 5 separate datasets. All datasets applied microarrays for transcriptomic analysis. The datasets examined peripheral blood mononuclear cells (*N* = 4) and whole blood (*N* = 1). The characteristics of included early dysglycemia datasets are further described in Table S4.

### Signal homogeneity assessment

To ensure that DEG datasets within AP-naive FEP and non-psychiatrically ill early dysglycemia groups are homogeneous and comparable with one another, we evaluated the concordance of each gene. Among AP-naive FEP studies which contained 22,008 genes in total, 18,357 (83.3%) genes exhibited concordant effect size, 3420 (15.5%) genes were conditionally concordant if filtered by *p* < 0.05, and only 231 (1.0%) genes were discordant. Among early dysglycemia studies containing a total of 38,418 genes, 12,241 (31.9%) genes showed concordant signals across all studies, 25,561 (66.5%) genes were concordant in studies with *p* < 0.05, and only 616 (1.6%) genes were discordant. The low percentage of discordant genes among both the AP-naive FEP and early dysglycemia studies (1.0% and 1.6% respectively) supports an overall concordance of signals across studies and supports our combination of signals across studies (*i.e*., meta-analysis) to increase power. The results of the signal homogeneity assessment are detailed in Table S5.

### Common differentially expressed genes

Following meta-analyses and filtering for genes that were concordant or conditionally concordant for both AP-naive FEP and non-psychiatrically ill early dysglycemia based on signal homogeneity assessment, we identified 221 common DEGs shared between AP-naive FEP and non-psychiatrically ill early dysglycemia (*p* < 0.05). Of these genes, 47 were upregulated and 62 were downregulated in both databases. The full results of the meta-analysis are detailed in Table S5.

### Pathway enrichment analysis

Pathway analysis via Metascape demonstrated that, for the common DEGs between AP-naive FEP and non-psychiatrically ill early dysglycemia datasets, there were 17 significant pathways (*p* < 0.05) (Table 1). These pathways were primarily related to ER stress [protein folding, protein sumoylation, regulation of calcium ion transport, and regulation of microRNA (miRNA) transcription] and abnormal brain bioenergetics [regulation of fatty acid metabolic process, positive regulation of receptor signaling pathway via Janus kinase/signal transducer and activator of transcription (JAK/STAT), positive regulation of glucose import, and regulation of glial cell proliferation]. The full results of the pathway analysis are detailed in Table S6.

**Table 1:** Functional clusters enriched in the common signatures between AP-naive FEP and early dysglycemia (*p* < 0.05).

Pathway name	# Genes found	−Log(P-value)	Genes
Regulation of fatty acid metabolic process	6/15	3.460399803	ACADVL,AKT1,IL1B,PTGS2,SNCA,MID1IP1
Regulation of glial cell proliferation	4/8	2.86158745	TSPO,IL1B,PPP1CC,RNF10
Negative regulation of microtubule polymerization or depolymerization	4/9	2.631020976	DYRK1A,SNCA,KATNB1,MID1IP1
Negative regulation of fibroblast proliferation	4/9	2.631020976	EMD,MYC,MED25,PARP10
Negative regulation of catabolic process	15/96	2.575941533	AKT1,TSPO,CSNK2A1,EIF4G2,FYN,IL1B,IL10,YBX1,SNCA,VHL,OGT,USP8,DHX34,BSCL2,AZIN1
Endocytosis	16/109	2.431050442	CANX,CSNK1D,CTBP1,DNM2,FCN1,SNCA,PSTPIP1,MTMR6,PDLIM7,SCAMP1,PACSIN2,SNX10,SCYL2,RAB22A,OCIAD2,C9orf72
Regulation of calcium ion transport	10/54	−2.404312995	TSPO,F2R,FYN,PTGS2,SNCA,CXCR4,LILRA2,TRPV2,AHNAK,LILRA5
Positive regulation of cold-induced thermogenesis	6/24	2.265940912	ACSL1,LCN2,CXCR4,OGT,BSCL2,TRPV2
Detection of external stimulus	3/6	2.2217321	FYN,CXCR4,PITPNM1
Positive regulation of Rho protein signal transduction	3/7	2.001880207	F2R,AKAP13,ARHGEF3
Protein modification by small protein conjugation	22/187	−1.945960753	AKT1,FYN,NFE2L2,SKP2,UBE2H,VHL,TRIM26,CUL4A,UBE4A,RNF10,RBX1,HMG20B,RUSC1,RNF11,MYLIP,PIAS4,FEM1C,PELI1,IFIH1,TRIM4,NSMCE1,FBXO33
Protein folding	10/63	1.920788148	CANX,CSNK2A1,DNAJB1,DNAJC4,NKTR,VBP1,SDF2L1,PPIL3,UNC45A,TXNDC5
Protein sumoylation	4/14	1.85357116	HMG20B,PIAS4,IFIH1,NSMCE1
Regulation of miRNA transcription	4/14	1.85357116	FOS,IL10,MYC,NCOR2
Positive regulation of receptor signaling pathway via JAK-STAT	4/14	1.85357116	F2R,IL10,CRLF3,OCIAD2
Response to temperature stimulus	8/46	1.852476337	ACADVL,AKT1,FOS,DNAJB1,PTGS2,CXCR4,PSIP1,TRPV2
Positive regulation of glucose import	3/8	1.82085996	AKT1,NFE2L2,RNASEL

*AP*-*naive* *FEP* antipsychotic-naive first-episode psychosis.

### Prediction efficiency of biomarkers

To validate our use of a meta-analytic approach to identify genes that may underly early dysglycemia in AP-naive FEP, we used two machine learning models (glmnet and random forest models) to test whether genes from the meta-analysis demonstrate predictive accuracy as biomarkers for illness status. Due to raw data unavailability of AP-naive FEP studies, we examined the possibility of predicting non-psychiatrically ill early dysglycemia status using our identified gene list. The glmnet and random forest models achieved promising average prediction accuracies of 0.71 and 0.73, respectively (Table S7). The final fitted glmnet model selected 88 genes as informative biomarkers for predicting non-psychiatrically ill early dysglycemia status. The top 10 genes with greatest magnitude of effect size were LILRA2, PPIL3, FYN, RUSC1, LCN2, PACSIN2, GALC, CHST7, TRIM26, and SDF2L1. The top 10 most important genes identified by the random forest model were COX4I1, LILRA2, CRLF3, TRIM26, EPSTI1, F13A1, PCMTD1, SH2D3C, FYN, and CCDC90B. Interestingly, the genes FYN, LILRA2, and TRIM26 were identified among the top 10 genes by both the glmnet and random forest models, which not only suggests these three genes as potentially important predictive biomarkers, but also validates the use of our meta-analytic approach to identify putative biomarker genes. The output of glmnet and random forest models are found in Tables S8 and S9 respectively.

### iLINCS identification of drug treatments

iLINCS is a web-based platform for analysis of omic signatures [37]. It holds an online library of systematically generated gene signatures, including transcriptomic profiles of over 40,000 drugs in various cell lines that represent gene expression changes in response to drug treatments. Considering that iLINCS contains established drug gene signatures, it can be used to identify putative drug candidates by searching for drugs within the library that have reverse signatures to the disease signatures. This approach has been used in several prior studies to identify putative drug candidates [28, 38–40].

We inputted the upregulated and downregulated genes that were common between AP-naive FEP and non-psychiatrically ill early dysglycemia into iLINCS, identifying 9 FDA-approved drugs with discordant gene signatures (Table 2). Of the identified drugs, the diabetes drug metformin [41–43] has been tested in the context of PSDs, with evidence showing that metformin ameliorates metabolic disturbances [42, 44–48]. Our results suggest that the agents we have identified may correct the overlapping gene expression changes of early dysglycemia and AP-naive FEP and hence represent putative treatments for intrinsic dysglycemia in PSDs. Importantly, based on these findings, we posit the presence of specific biological pathways that are shared between dysglycemia and PSDs that provide the mechanisms of action for the identified agents.

**Table 2:** FDA-approved drugs with signatures discordant to the signatures of AP-naive FEP and early dysglycemia.

Perturbagen	Canonical Mechanism of Action	Anatomical Therapeutic Chemical First Level Classification	Studies in PSDs
Chlorpropamide	Bind to ATP-sensitive potassium channels on pancreas, leading to insulin secretion	Alimentary tract and metabolism	-
Clobetasol	Bind to glucocorticoid receptors	Dermatologicals	-
Daunorubicin	DNA topoisomerase inhibitor	Antineoplastic and immunomodulating agents	-
Flunisolide	Glucocorticoid receptor agonist	Respiratory system	-
Irinotecan	DNA topoisomerase inhibitor	Antineoplastic and immunomodulating agents	-
Medrysone	Glucocorticoid receptor agonist	Sensory Organs	-
Mestranol	Estrogen receptor agonist	Not available	-
Metformin	Inhibits mitohcondrial complex 1 in the liver	Alimentary tract and metabolism	de Silva et al., 2016; Siskind et al., 2016; Praharaj et al., 2011
Tamoxifen	Estrogen receptor competitive inhibitor; protein Kinase C inhibitor; sex hormone-binding globulin inducer	Antineoplastic and immunomodulating agents	-

The canonical mechanism of action was referenced from DrugBank (https://go.drugbank.com/) and the classification from Anatomical Therapeutic Chemical (https://www.whocc.no/atc_ddd_index/). Studies examining these agents as a potential therapeutic for psychopathology or metabolic dysfunction in PSDs are indicated on the right.

*AP-naive FEP* antipsychotic-naive first-episode psychosis, *PSDs* psychosis spectrum disorders

## Discussion

Increasing evidence suggests that dysglycemia is intrinsic to the pathophysiology of PSDs and therefore occurs independently of the metabolic liabilities resulting from AP treatment [49]. An important clue supporting this observation is that markers of early dysglycemia, including insulin resistance and impaired glucose tolerance, are observed in early psychosis patients who are AP-naive [9]. Nonetheless, the biological mechanisms underlying intrinsic dysglycemia remain largely unknown. This raises the question of whether there are shared pathophysiological mechanisms between PSDs and early dysglycemia in non-psychiatrically ill patients at the gene expression level. To identify these mechanisms, we examined whether AP-naive FEP and non-psychiatrically ill early dysglycemia patients present with an overlap in DEGs. We found 221 common DEGs, suggesting that PSDs and early dysglycemia indeed share common gene expression signatures. These findings extend previous work examining the genetic links between PSDs and T2D [12–14] by demonstrating that an overlap exists at the gene expression level. The overlapping gene expression signatures between AP-naive FEP and early dysglycemia potentially represent gene expression changes endogenous to PSDs that are responsible for producing dysglycemia independent of AP treatment. Consequently, our findings suggest that dysglycemia is intrinsic to PSDs, and may be further exacerbated by AP treatment.

**Fig. 3:**
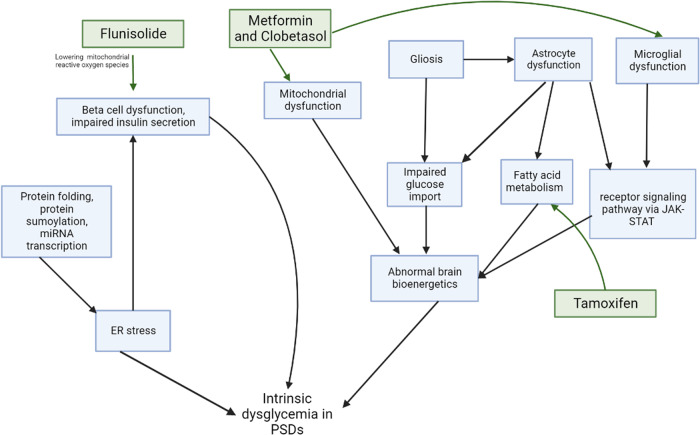
Pathways that are potentially impacted by drugs. The pathways associated with dysglycemia in psychosis spectrum disorders (PSDs) and the possible mechanisms by which flunisolide, metformin, clobetasol and tamoxifen may reverse dysglycemia.

The common DEGs between AP-naive FEP and non-psychiatrically ill early dysglycemia revealed potential mechanisms for intrinsic dysglycemia in PSDs (Fig. 3). Notably, our pathway analyses demonstrated processes suggestive of abnormal glucose bioenergetics in the brain, including regulation of glucose transmembrane transport. Glucose represents the primary source of energy for the brain and is transported across the blood brain barrier from peripheral circulation to astrocytes via glucose transporter 1 (GLUT1) [50]. Glucose then undergoes glycolysis into lactate, which not only represents a fuel source for neurons, but also mediates regulation of whole-body glucose homeostasis [50, 51]. Specifically, ATP produced from metabolizing lactate activates ATP-sensitive potassium (K_ATP_) channels in hypothalamic neurons. This, in turn, signals the liver to reduce hepatic glucose production, a process termed glucose sensing [50, 51]. Thus, dysfunctions in hypothalamic bioenergetics and glucose sensing may underly dysglycemia intrinsic to PSDs. Accordingly, patients with PSDs demonstrate elevated brain lactate levels, a marker of deranged bioenergetics [52, 53].

Additional bioenergetic abnormalities may contribute to these intrinsic metabolic disturbances including mitochondrial dysfunction. Indeed, mitochondrial markers are observed throughout the progressive stages of insulin resistance and glucose dysregulation that ultimately culminate in T2D [54], as well as in the blood and brain of schizophrenia patients [52, 53, 55–57]. This suggests that mitochondrial dysfunction may directly hamper oxidative phosphorylation and ATP production in neurons, thereby deranging brain bioenergetics and glucose sensing to induce intrinsic, systemic dysglycemia.

A key contributor to abnormal brain bioenergetics in PSDs may be gliosis, consistent with our findings implicating glial proliferation as another top pathway shared between AP-naive FEP and non-psychiatrically ill early dysglycemia. Gliosis, which is characterized by proliferation, neuroinflammation, and morphological transformation of astrocytes and microglia, may dysregulate brain bioenergetics by impairing glial modulation of hypothalamic neurons required for metabolic control [58]. Astrocytes play crucial roles in glucose uptake into the brain, lactate production, and glucose sensing [50, 51]. In line with this, astrocytic dysfunction results in a reduction of glucose uptake into the brain, impairing peripheral glucose tolerance [59]. Glial cells also have important roles in the regulation of lipid homeostasis and fatty acid synthesis in the central nervous system [60], consistent with the enrichment of regulation of fatty acid metabolism in the pathway analysis. Indeed, dysfunction of astrocytes has been linked to disorders of central and peripheral lipid and glucose metabolism [60, 61]. Alterations in the expression of the lipid chaperone fatty acid binding protein 7 (FABP7), which is expressed primarily in astrocytes in the brain [62], have been found in the brains of schizophrenia patients [63].

In addition, activation and proliferation of microglia and astrocytes result in an upregulation of proinflammatory cytokines and pathways in the hypothalamus, which exacerbate gliosis and induce peripheral dysglycemia [64]. Upregulation of inflammatory IKKβ/NF-κB signaling in rodents also impairs astrocyte process remodeling which is expected to impair astrocytic regulation of neurons, and in turn, reduce glucose tolerance [65]. Conversely, downregulating IKKβ/NF-κB signaling diminishes hypothalamic inflammation, gliosis, and peripheral glucose intolerance [66–70]. Furthermore, JAK/STAT signaling, which was implicated by the pathway analysis, may contribute to neuroinflammation in PSDs [71] through overactivation of astrocytes and microglia [72]. The JAK/STAT pathway is additionally involved in regulation of glucose homeostasis through various mechanisms including effects on pancreatic β-cell function and hepatic glucose metabolism [73]. Taken together, gliosis along with associated abnormalities in brain bioenergetics, neuroinflammation, and JAK/STAT signaling may contribute to intrinsic dysglycemia in PSDs. Nonetheless, while gliosis has been associated with impaired glucose tolerance and insulin resistance in humans [74, 75], the precise relationships between gliosis and intrinsic dysglycemia of PSDs remain unclear [76], requiring future investigation.

The overlap between AP-naive FEP and non-psychiatrically ill early dysglycemia was also characterized by pathways related to ER stress, a state characterized by an accumulation of unfolded and misfolded proteins. Specifically, our analyses implicated pathways relating to protein folding, protein sumoylation, regulation of calcium ion transport, and microRNA (miRNA) transcription. Sumoylation, a post-translational protein modification, modulates the function of transcription factor, X box-binding protein 1 (XBP1), which in turn regulates gene expression of proteins involved in ER stress [77]. Just as importantly, XBP1 is associated with hepatic insulin resistance via regulation of gene expression involved in hepatic glucose production [78]. Calcium ions are important players in ER stress-mediated cellular apoptosis, as well as quality control via chaperones, which ensure proper folding of proteins [79]. MiRNAs are non-coding RNAs that may be linked to ER stress through cell survival or apoptotic mechanisms [80]. Chronic ER stress and abnormal unfolded protein response activation have been implicated in both PSDs [81–84] and dysglycemia [78, 85]. For example, in the dorsolateral prefrontal cortex of schizophrenia patients, aberrant expression of proteins involved in the unfolded protein response has been postulated to contribute to aberrant neurotransmission [83, 84]. In the periphery, ER stress plays important roles in pancreatic β-cells which may also contribute to dysglycemia. Indeed, sustained ER stress has been implicated in β-cell dysfunction and impaired insulin secretion in T2D [85]. Additionally, genes involved in ER stress are implicated in abnormal glycemic control, including PERK, JNK and XBP1 [86]. Of these, genetic variants of XBP1 have also been linked to schizophrenia [87, 88]. Taken together, ER stress may represent a potential mechanism contributing to intrinsic dysglycemia in PSDs.

Our findings have significant implications by demonstrating promising prediction accuracies of the meta-analyzed genes. By using two machine learning models (glmnet and random forest models), we demonstrated that the DEGs identified by our meta-analysis among non-psychiatrically ill early dysglycemia studies were able to predict early dysglycemia status with reasonable accuracy. In particular, the genes FYN, LILRA2, and TRIM26 were implicated as top 10 biomarker genes by both the glmnet and random forest models. Of these, FYN has previously been suggested to play a role in glucose regulation in preclinical models [89–91], which supports the clinical validity of this gene as a potential predictive biomarker. While it was not possible to apply the prediction models on the AP-naive FEP datasets due to raw data unavailability, future studies would benefit from applying similar prediction models to determine biomarker genes that may differentiate the presence or absence of a dysglycemic phenotype in AP-naive FEP patients.

Our findings hold additional significant clinical and translational value by identifying candidate pharmacological treatments for intrinsic dysglycemia in PSDs. We found that the pharmacological agents identified in our iLINCs analysis have gene expression patterns discordant to the common gene expression signatures of AP-naive FEP and early dysglycemia in non-psychiatrically ill individuals. This suggests that these drugs may correct the common gene expression changes and, in effect, treat intrinsic dysglycemia in PSDs. Notably, we identified two antidiabetic medications, chlorpropamide and metformin. The sulfonylurea chlorpropamide has been withdrawn from the market, and other sulfonylureas are not recommended as first-line agents due to adverse events [92]. On the other hand, the identification of metformin in the iLINCS analysis validates our results since metformin has independently been identified as a therapeutic agent for ameliorating PSD-induced metabolic dysfunction. As a first-line pharmacological treatment for T2D, metformin is also recommended off-indication in national guidelines to mitigate or prevent AP-induced weight gain [92, 93]. Accordingly, metformin treatment improved glycemic parameters across randomized-controlled trials (RCTs) and meta-analyses in patients with schizophrenia [42, 44, 45, 94]. Several of these studies specifically tested metformin in FEP patients, demonstrating the beneficial effects of early treatment in attenuating AP-induced dysglycemia [44, 46–48]. Our results suggest that early metformin treatment could additionally address intrinsic metabolic alterations, dually addressing two significant factors (intrinsic and APs) that contribute to metabolic dysfunction in this population.

Some of the iLINCS-identified drugs may impact the pathways associated with intrinsic dysglycemia in PSDs (Fig. 3). Notably, metformin reduces microglial activation [95, 96] and improves mitochondrial function [97], supporting the proposition that metformin may correct the gene expression signatures of intrinsic dysglycemia in PSDs. There is also some evidence that tamoxifen may oppose changes in the identified pathways. Specifically, tamoxifen alters fatty acid metabolism by reducing fatty acid synthase in the liver and hypothalamus [98], lowering liver lipid accumulation, and improving glucose tolerance in preclinical studies [99]. However, hyperglycemia and increased risk of diabetes associated with tamoxifen treatment in patients [100, 101] limit the clinical utility of tamoxifen in treating dysglycemia in PSDs. Another drug that shows evidence in potentially reversing the identified pathways is clobetasol. In mice, clobetasol can lower pro-inflammatory microglial signaling and reverse markers of mitochondrial dysfunction, such as mitochondrial fission [102]. However, there is also evidence showing that glucocorticoids such as clobetasol can induce hyperglycemia [103]. Finally, flunisolide may reverse the identified pathways by diminishing cytotoxic mitochondrial reactive oxygen species [104], consequently lowering oxidative stress associated with insulin resistance [105].

Our findings may provide future insight into premorbid cognitive dysfunction in PSDs. It is well established that PSDs are characterized by early premorbid cognitive dysfunction [106]. Patients with PSDs also demonstrate glucose dysregulation early in the illness course. Given associations between T2D and insulin resistance with worse cognitive impairments in the general population and in schizophrenia [17, 18], we speculate that the pathophysiology underlying premorbid cognitive dysfunction and intrinsic dysglycemia overlap in PSDs. To this point, accumulating evidence suggests that brain defects in glucose and lactate utilization as well as mitochondrial dysfunction may be linked to cognitive dysfunction [38, 53, 107–109]. Thus, we posit that brain bioenergetic defects could dually explain intrinsic dysglycemia and premorbid cognitive dysfunction in PSDs. If true, this would have implications for the identified candidate drug treatments in this study to potentially target both intrinsic dysglycemia and cognitive dysfunction in PSDs.

Although our findings build on growing literature supporting intrinsic metabolic dysfunction in PSDs and identify possible pathways explaining these associations, some limitations should be noted. The pathway results demonstrated blood-related processes that may be less specific or informative to brain mechanisms, which likely resulted from the high proportion of FEP studies examining blood-derived tissue. Nonetheless, we observed glial cell proliferation, a neurobiological process, amongst our pathway results, which is consistent with the transcriptomic overlap reported between peripheral blood and the brain [19, 20] to support our examination of peripheral tissue. Furthermore, post-mortem brain samples are rare in AP-naive FEP patients, who tend to be young. Available post-mortem brain samples of patients with PSDs tend to be confounded by years of AP exposure and illness-related lifestyle factors and would thus significantly hinder examination of illness intrinsic gene expression changes. As an additional limitation to our study, the pathway and iLINCS analyses are biased towards extensively studied biological processes. The multiple hits in our iLINCS analysis for anticancer drugs likely reflect the well-studied nature of cancer. An additional explanation for the anticancer drugs may be that a potential link exists between schizophrenia and cancer. Currently, there is mixed literature on the incidence of cancer among schizophrenia patients, including increased, decreased, or no difference in cancer incidence [110]. In support of decreased incidence of cancer in schizophrenia patients, one theory asserts that, despite increased risk factors such as smoking and substance abuse, opposing activities of the adenosine system between schizophrenia and cancer may drive this dichotomous relationship [110]. Hypofunction of the adenosine system observed in schizophrenia may be protective against the increased adenosine metabolism found in cancer [110]. Additionally, adenosine signaling also plays a role in glucose metabolism and insulin secretion [111], which further supports a link between PSDs and dysglycemia. In contrast, cancer signaling pathways have been reported to be elevated in schizophrenia patients [112]. This link may explain the multiple anticancer drugs found in our iLINCS analysis, however, further research is needed to more clearly understand the relationship between schizophrenia and cancer. Nonetheless, the presence of antidiabetic agents in the iLINCS analysis, which would be expected to correct metabolic abnormalities in PSDs, supports the validity of our results. Furthermore, for the AP-naive FEP studies, we used reported differential gene expression results instead of conducting differential expression analysis on raw expression data; this limitation is due to data unavailability from the original studies. This may have introduced variations in the transcriptomic signatures, as the data may not have been processed and analyzed consistently across the studies. Additional potential confounders include demographic factors such as age and sex, as few studies did not report these values and may not have matched the case and unaffected comparison groups to these factors.

Collectively, we confirm emerging data supporting shared intrinsic disease pathways between PSDs and dysglycemia at a gene expression level. Dysregulations in ER stress and brain bioenergetics may contribute to the pathophysiology of PSDs and also contribute to dysglycemia in PSDs. Therefore, dysglycemia may be intrinsic to PSDs, much like the psychiatric symptoms that define these illnesses. It would be important to test the antidiabetic agent metformin as a potential treatment for dually mitigating intrinsic and AP-induced dysglycemia in PSDs at least in a subgroup of individuals. The ability to use our approaches to both better define subtypes of PSDs, as well as more precisely target important yet underappreciated metabolic aspects of these illnesses may have implications on cardiometabolic health and potentially aspects of psychopathology.

### Supplementary information

Tables S1-S4, S7

Table S5

Table S6

Table S8

Table S9

## Data Availability

No new primary data were collected or analyzed in this study. The results of all analyses are available within the main manuscript or as supplementary files. The GitHub link containing all scripts used in the analyses is available under the Methods section. Figure 3 was created using BioRender (BioRender.com).
