# Supplementary material for: Glucose dysregulation in antipsychotic-naive first-episode psychosis: in silico exploration of gene expression signatures
Source: Transl Psychiatry. 2024 Jan 10;14:19. doi: 10.1038/s41398-023-02716-8 (PMC10781725; doi:10.1038/s41398-023-02716-8)
Supplement: Supplementary file 1 — Tables S1-S4, S7 [file 41398_2023_2716_MOESM1_ESM.docx]

**Table S1.** Search string used for antipsychotic-naïve first episode psychosis studies in Ovid MEDLINE: Epub Ahead of Print, In-Process & Other Non-Indexed Citations, Ovid MEDLINE® Daily and Ovid MEDLINE® 1946-Present

| **#** | **Searches** |
| --- | --- |
| 1 | antipsychotic naive.mp. |
| 2 | antipsychotic free.mp. |
| 3 | drug naive.mp. |
| 4 | drug free.mp. |
| 5 | neuroleptic naive.mp. |
| 6 | neuroleptic free.mp. |
| 7 | never treated.mp. |
| 8 | never medicated.mp. |
| 9 | untreated.mp. |
| 10 | unmedicated.mp. |
| 11 | first episode.mp. |
| 12 | exp Schizophrenia/ or schizophren*.mp. |
| 13 | psychos#s.mp. |
| 14 | exp Transcriptome/ or transcriptom*.mp. |
| 15 | RNA seq*.mp. |
| 16 | RNAseq.mp. |
| 17 | RNA expression.mp. |
| 18 | RNA profil*.mp. |
| 19 | mRNA-seq*.mp. |
| 20 | mRNA expression.mp. |
| 21 | mRNA profil*.mp. |
| 22 | microarray*.mp. |
| 23 | gene expression.mp. |
| 24 | exp Sequence Analysis, RNA/ or exp RNA-Seq/ or exp Gene Expression Profiling/ |
| 25 | exp Microarray Analysis/ |
| 26 | microarray analysis/ or oligonucleotide array sequence analysis/ or tissue array analysis/ |
| 27 | treatment naive.mp. |
| 28 | treatment free.mp. |
| 29 | medication naive.mp. |
| 30 | medication free.mp. |
| 31 | 1 or 2 or 3 or 4 or 5 or 6 or 7 or 8 or 9 or 10 or 11 or 27 or 28 or 29 or 30 |
| 32 | 12 or 13 |
| 33 | 14 or 15 or 16 or 17 or 18 or 19 or 20 or 21 or 22 or 23 or 24 or 25 or 26 |
| 34 | 31 and 32 and 33 |

**Table S2.** Search string used for early dysglycemia studies in Ovid MEDLINE: Epub Ahead of Print, In-Process & Other Non-Indexed Citations, Ovid MEDLINE® Daily and Ovid MEDLINE® 1946-Present

| # | **Searches** |
| --- | --- |
| 1 | prediabetes.mp. or exp Prediabetic State/ |
| 2 | exp Glucose Intolerance/ or IGT.mp. |
| 3 | IFG.mp. |
| 4 | IPG.mp. |
| 5 | impaired fasting glucose.mp. |
| 6 | impaired glucose tolerance.mp. |
| 7 | impaired plasma glucose.mp. |
| 8 | hyperglycaemia.mp. |
| 9 | hyperglycemia.mp. or exp Hyperglycemia/ |
| 10 | exp Insulin Resistance/ or insulin resistan*.mp. |
| 11 | borderline diabetes.mp. |
| 12 | prediabetic state.mp. |
| 13 | exp Transcriptome/ or exp Gene Expression Profiling/ or transcriptom*.mp. |
| 14 | exp Sequence Analysis, RNA/ or RNA seq*.mp. |
| 15 | RNA expression.mp. |
| 16 | RNA profil*.mp. |
| 17 | mRNA-seq*.mp. |
| 18 | mRNA expression.mp. |
| 19 | mRNA profil*.mp. |
| 20 | exp Microarray Analysis/ or microarray*.mp. or exp Oligonucleotide Array Sequence Analysis/ |
| 21 | gene expression.mp. or exp Gene Expression/ |
| 22 | exp Blood/ or blood.mp. |
| 23 | exp Monocytes/ or exp Leukocytes, Mononuclear/ or PBMC*.mp. |
| 24 | peripheral blood mononuclear cell*.mp. |
| 25 | PBL*.mp. |
| 26 | peripheral blood leukocyte*.mp. |
| 27 | leukocyte*.mp. |
| 28 | blood-based.mp. |
| 29 | monocyte*.mp. |
| 30 | lymphocyte.mp. or exp Lymphocytes/ |
| 31 | 1 or 2 or 3 or 4 or 5 or 6 or 7 or 8 or 9 or 10 or 11 or 12 |
| 32 | 13 or 14 or 15 or 16 or 17 or 18 or 19 or 20 or 21 |
| 33 | 22 or 23 or 24 or 25 or 26 or 27 or 28 or 29 or 30 |
| 34 | 31 and 32 and 33 |
| 35 | limit 34 to (english language and humans) |
| 36 | exp Diabetes Mellitus, Type 2/ or diabetes.mp. or exp Diabetes Mellitus/ |
| 37 | 31 or 36 |
| 38 | 32 and 33 and 37 |
| 39 | limit 38 to (english language and humans) |

**Table S3.** Characteristics of included AP-naïve FEP studies.

| **Study** | **N** | |  | **Age, BMI (Mean (SD)),**  **Sex (% Female)** | | **Cases Psychiatric Diagnosis** | **Tissue** | **Platform Description** | **Reported Differential Expression Data** |
| --- | --- | --- | --- | --- | --- | --- | --- | --- | --- |
|  | **AP-naïve FEP** | **CTRL** |  | **AP-naïve FEP** | **CTRL** |  |  |  |  |
| Leirer et al., 2019 [1] | 18 | 149 |  | Not reported for AP-naïve subgroup | Age= 29.87 (10.53)  %F= 42.3  BMI not reported | DSM-IV Criteria SCZ; ICD-10 criteria for non-affective or affective psychosis | Whole Blood | Microarray: Illumina HT-12 V4 bead-chips | Complete data; 4063 genes |
| Sainz et al., 2013 [2] | 36 | 40 |  | Age= 29.7 (9.7)  %F= 47.4%  BMI not reported | Age= 33 (8.3)  %F= 47.5:  BMI not reported | SCZ; SZA; SZPD; BPD; Delusional Disorder | Whole Blood | RNA Sequencing | Complete data; 22289 genes |
| Xavier et al., 2020 [3] | 55 | 57 |  | Age= 25.7 (7.4)  %F= 35.7  BMI not reported | Age= 25.95 (7.42)  %F= 56.1  BMI not reported | DSM-IV Criteria for SCZ; SZPD; BPD | Whole Blood | Microarray; Illumina HumanHT-12 v4 Expression BeadChip | Complete data; 5935 genes |
| Kumarasinghe et al., 2013 [4] | 10 | 11 |  | Age= 36.1 (14.8)  %F= 20 | Age= 35.7 (14.5)  %F= 54.5 | DSM-IV Criteria for SCZ | PBMCs | Microarray; Illumina HT-12_V3 beadchips | Partial data; 625 significant genes |
| Gasso et al., 2017 Fibroblasts [5] | 11 | 9 |  | Age= 23.5 (1.1)  BMI= 21.3 (1.1)  %F= 45.5 | Age= 22.8 (0.7)  BMI= 21.7 (0.5)  %F= 44.5 | DSM-IV Criteria for SCZ | Fibroblasts | Microarray; Affymetrix GeneChip HG U219 Array Plate | Partial data; 179 significant genes |
| Gasso et al., 2017 Lymphoblastoid [5] | 11 | 9 |  | Age= 23.5 (1.1)  BMI= 21.3 (1.1)  %F= 45.5 | Age= 22.8 (0.7)  BMI= 21.7 (0.5)  %F= 44.5 | DSM-IV Criteria for SCZ | Lymphoblastoid | Microarray; Affymetrix GeneChip HG U219 Array Plate | Partial data; 817 significant genes |

CTRL=controls, AP=antipsychotic, FEP=first episode psychosis, PBMCs=peripheral blood mononuclear cells, DSM-IV= Diagnostic and Statistical Manual of Mental Disorders, 4th Edition, ICD=International Classification of Diseases, SCZ=schizophrenia, SZA=schizoaffective disorder, SZPD=schizophreniform disorder, BPD=brief psychotic disorder.

**Table S4.** Characteristics of included early dysglycemia studies.

| **Study, GEO ID** |  | **N** | |  | **Glycemic Markers, Mean (SD)** | |  | **Age, BMI (Mean (SD)),**  **Sex (% Female)** | |  | **Tissue** | **Platform Description** |
| --- | --- | --- | --- | --- | --- | --- | --- | --- | --- | --- | --- | --- |
|  |  | **Early dysglycemia** | **CTRL** |  | **Early dysglycemia** | **CTRL** |  | **Early dysglycemia** | **CTRL** |  |  |  |
| Berry 2018 [6]  GSE101931 |  | 5 | 5 |  | FBG= 101.8 (9.7) mg/dl  HbA1c= 6 (1.3) % | FBG= 82.8 (9.7) mg/dl  HbA1c= 5.7 (0.3) % |  | Age= 33 (9)  BMI= 32.1 (4.0)  Sex not reported | Age= 35 (9)  BMI= 29.4 (5.2)  Sex not reported |  | PBMCs | Microarray; Illumina HumanHT-12 V4.0 expression beadchip |
| Karolina 2011 [7]  GSE21321 |  | 7 | 10 |  | FBG= 6.4 (0.1) mmol/L | FBG= 4.7 (0.7) mmol/L |  | Age= 49.0 (7.6)  BMI= 24.1 (2.7)  %F= 0 | Age= 46.3 (7.5)  BMI= 22.4 (2.3)  %F= 0 |  | Whole blood | Microarray; Illumina HumanRef-8 v3.0 expression beadchip |
| Mallu 2017 [8]  GSE153837 |  | 9 | 6 |  | FBG= 111.4 (2.82) mg/dl  OGTT 2hr Glucose= 143.1 (12.9) | FBG= 89.5 (2.68) mg/dl  OGTT 2hr Glucose= 98.0 (10.11) |  | Age= 32.62 (2.98)  BMI= 28.21 (1.49)  Sex not reported | Age= 31.67 (0.84)  BMI= 27.68 (1.52)  Sex not reported |  | PBMCs | Microarray; Illumina HumanHT-12 V4.0 expression beadchip |
| Matone 2017 [9] Low BMI group  GSE87005 |  | 10 | 10 |  | HOMA-IR= 4.0 (2.8) | HOMA-IR= 0.5 (0.1) |  | Age= 33.9 (6.1)  BMI= 22.2 (1.6)  %F= 30 | Age= 40.2 (11.3)  BMI= 22.5 (2.2)  %F= 30 |  | PBMCs | Microarray; Agilent-014850 Whole Human Genome Microarray 4x44K G4112F |
| Matone 2017 [9]  High BMI group  GSE87005 |  | 10 | 10 |  | HOMA-IR= 3.1 (0.8) | HOMA-IR= 0.8 (0.3) |  | Age= 38.1 (6.9)  BMI= 27.9 (2.7)  %F= 30 | Age= 36.7 (4.7)  BMI= 27.7 (2.5)  %F= 40 |  | PBMCs | Microarray; Agilent-014850 Whole Human Genome Microarray 4x44K G4112F |

CTRL=controls, PBMCs=peripheral blood mononuclear cells; FBG=fasting blood glucose; HbA1c=hemoglobin A1C; GEO=Gene Expression Omnibus; OGTT=oral glucose tolerance test; SD=standard deviation, HOMA-IR=homeostatic model of insulin resistance.

**Table S7.** Prediction accuracies of the meta-analyzed non-psychiatrically ill dysglycemia datasets in predicting early dysglycemia status using glmnet and random forest models. At each iteration, each of the five datasets were used as test data, while the remaining four datasets were combined by meta-analysis and used to train the model.

| Test data | GSE101931 | GSE21321 | GSE153837 | GSE87005_1 | GSE87005_2 | Average |
| --- | --- | --- | --- | --- | --- | --- |
| glmnet | 0.70 | 0.73 | 0.87 | 0.65 | 0.60 | 0.71 |
| Random Forest | 0.70 | 0.80 | 0.67 | 0.65 | 0.85 | 0.73 |

**References**

1. Leirer, D.J., et al., *Differential gene expression analysis in blood of first episode psychosis patients.* Schizophr Res, 2019. **209**: p. 88-97.

2. Sainz, J., et al., *Inflammatory and immune response genes have significantly altered expression in schizophrenia.* Mol Psychiatry, 2013. **18**(10): p. 1056-7.

3. Xavier, G., et al., *Blood gene expression changes after Risperidone treatment in an antipsychotic-naive cohort of first episode of psychosis patients.* Schizophr Res, 2020. **220**: p. 285-286.

4. Kumarasinghe, N., et al., *Gene expression profiling in treatment-naive schizophrenia patients identifies abnormalities in biological pathways involving AKT1 that are corrected by antipsychotic medication.* Int J Neuropsychopharmacol, 2013. **16**(7): p. 1483-503.

5. Gassó, P., et al., *Microarray gene-expression study in fibroblast and lymphoblastoid cell lines from antipsychotic-naïve first-episode schizophrenia patients.* J Psychiatr Res, 2017. **95**: p. 91-101.

6. Berry, N.T., M. Hubal, and L. Wideman, *The effects of an acute exercise bout on GH and IGF-1 in prediabetic and healthy African Americans: A pilot study investigating gene expression.* PLoS One, 2018. **13**(1): p. e0191331.

7. Karolina, D.S., et al., *MicroRNA 144 impairs insulin signaling by inhibiting the expression of insulin receptor substrate 1 in type 2 diabetes mellitus.* PLoS One, 2011. **6**(8): p. e22839.

8. Mallu, A.C.T., et al., *Prediabetes uncovers differential gene expression at fasting and in response to oral glucose load in immune cells.* Clin Nutr, 2021. **40**(3): p. 1247-1259.

9. Matone, A., et al., *Identification of an early transcriptomic signature of insulin resistance and related diseases in lymphomonocytes of healthy subjects.* PLoS One, 2017. **12**(8): p. e0182559.
